# First Steps into Language? Examining the Specific Longitudinal Relations between Walking, Exploration and Linguistic Skills

**DOI:** 10.3389/fpsyg.2016.01458

**Published:** 2016-09-27

**Authors:** Ora Oudgenoeg-Paz, M(Chiel). J. M. Volman, Paul P. M. Leseman

**Affiliations:** Department of Child, Family and Education Studies, Utrecht UniversityUtrecht, Netherlands

**Keywords:** motor milestones, independent walking, exploration, spatial language, language development, embodiment

## Abstract

Recent empirical evidence demonstrates relationships between motor and language development that are partially mediated by exploration. This is in line with the embodied cognition approach to development that views language as grounded in real-life sensorimotor interactions with the environment. This view implies that the relations between motor and linguistic skills should be specific. Moreover, as motor development *initially* changes the possibilities children have to explore the environment, initial relations between motor and linguistic skills should become weaker over time. Empirical evidence pertaining to the duration and specificity of these relations is still lacking. The current study investigated longitudinal relations between attainment of walking and the development of several linguistic skills, and tested whether exploration through self-locomotion mediated these relations. Linguistic skills were measured at age 43 months, which is later than the age used in previous studies. Three hypotheses were tested: (1) the relations between walking and language found at younger ages will decrease over time (2) exploration through self-locomotion will remain an important predictor of spatial language (3) no relation will be found between walking, exploration and the use of grammatical and lexical categories and between exploration and general vocabulary. Thirty-one Dutch children took part in a longitudinal study. Parents reported about age of attainment of walking. Exploration through self-locomotion was measured using observations of play with a standard set of toys at age 20 months. Receptive vocabulary, spatial language and use of grammatical and lexical categories were measured at age 43 months using (standard) tests. Results reveal that age of walking does not directly predict spatial language at age 43 months. Exploration through self-locomotion does significantly and completely mediate the indirect effect of age of walking on spatial language. Moreover, neither age of walking nor exploration predict general vocabulary and the use of grammatical and lexical categories. Results support the idea that the initial relations between motor development and linguistic skills decrease over time and that these relations are specific and intrinsically dependent on the information children pick up through the execution of specific motor activities.

## Introduction

In recent years, an increasing number of theoretical and empirical papers have addressed the link between motor development and development in other domains. One of the central theoretical approaches stressing the role of movement and motor control in cognitive-linguistic development is the embodied cognition approach (e.g., Hockema and Smith, 2009; Iverson, 2010). According to this approach, cognitive skills (including linguistic skills) are softly assembled in real-time from elementary perception-action processes that are rooted in concrete real-life interactions (Thelen and Smith, 1994). Development of new motor skills, such as sitting, crawling and walking opens the door to new ways of interacting with the environment and fundamentally alters children’s sensorimotor experiences by bringing new possibilities of exploring the environment (Gibson, 1988; Thelen and Smith, 1994; Soska and Adolph, 2014). The increased possibilities for exploring objects, relations between objects, and spatial layouts provide the cognitive basis for language learning. Therefore, the development of motor skills is expected to predict linguistic advances. The situated nature of cognitive skills (including linguistic skills) suggests highly specific relations between what is explored and the cognitive-linguistic skills that are grounded in these specific sensorimotor interactions. For example, exploring objects that can be stacked is expected to be related to the development of spatial concepts and related prepositions such as ‘*on’* and ‘*under,’* but not to the learning of food names or grammatical skills such as verb inflection. While in recent years increasing evidence is reported for a link between the development of motor skills and language development (e.g., Oudgenoeg-Paz et al., 2012; Walle and Campos, 2014; Libertus and Violi, 2016), evidence regarding the *specific* and *meaning-intrinsic* nature of these links, how they influence cognitive-linguistic development over longer periods of time and the role of exploration as a mediating process between motor skill development and cognitive-linguistic outcomes is still scarce. The current study focuses on the specific links between attainment of motor milestones and the development of several linguistic skills. By doing so we aim to study not only which linguistic skills are related to motor development, but also which skills are *not*. The linguistic skills examined in the current study include general and spatial vocabulary and the use of grammatical and lexical categories (e.g., determiners, subjects). In addition, the current study also examines the long-term associations between motor milestone attainment and cognitive-linguistic skills. Previous studies demonstrated mainly relations in the short term, but it is not clear how stable these relations are when examined longitudinally over longer periods of time. Finally, the current study examines the role of exploration behavior as a mediator of the relation between motor milestones attainment and linguistic skills.

In line with the embodied cognition approach, Hockema and Smith (2009) describe linguistic development as composed of recurrent outside-in processes (children perceive information from the environment) and inside-out processes (children act on the environment). For example, when learning semantic categories, children may draw on physical features of objects to categorize them (e.g., all round objects are balls), but children may also actively sort similar objects to the same spatial location, thus making it easier for them to perceive the similarities and construct a semantic category. This model of language learning is also in line with ecological psychology theory stressing the central role of recurrent perception-action processes in development. Children, on the one hand, perceive information to be processed, but on the other hand, they also act in their environment and thus change the information they can perceive (Gibson, 1979; Gibson and Pick, 2000). While the classical ecological approach does not mention cognition at all (Gibson, 1979), the embodied view of cognition as situated in the context of an agent interacting with the environment, is in full agreement with the notion of recurrent perception-action cycles in which increasingly complex affordances are detected and the skill to act upon these affordances is developed. This notion, that is central to the ecological approach, will here be referred to as exploration (Thelen and Smith, 1994; Gibson and Pick, 2000; Smith and Gasser, 2005). The knowledge about the world acquired through exploration forms the basis for the development of advanced cognitive (and linguistic) skills (Gibson and Pick, 2000; Smith and Gasser, 2005).

The attainment of motor milestones dramatically changes children’s exploration possibilities and is therefore expected to contribute to further advances in their linguistic development. A major milestone in motor development is the attainment of independent walking. Exploration of the environment through self-locomotion enables children first of all to explore more complex spatial relations that require taking different positions in space or moving objects to different places in space. The exploration of these spatial relations is expected to relate to more complex spatial language (e.g., the prepositions ‘behind,’ ‘between,’ ‘through’). Moreover, moving around initiates a shift from predominantly egocentric to allocentric view of spatial relations and therefore allows children to learn about different, dynamically changing perspectives of their environment. This shift has been related to advances in spatial cognition (Campos et al., 2000; Newcombe, 2002; Sheya and Smith, 2011). Thus, exploring objects while engaging in self-locomotion enables infants to experience the relationship between their own movement and changing position and the position and view of the object they are exploring (Thelen and Smith, 1994). In addition, walking enables children not only to carry objects from one location to another, but also to draw parents’ attention to their exploratory behavior and to the objects they are engaged with. This implies that children that are able to walk independently are more likely to receive linguistic input pertaining to their current focus of attention (Clearfield, 2011; Karasik et al., 2011). Thus, attainment of independent walking is expected to facilitate language acquisition in general, and the acquisition of spatial language in particular, because walking specifically enables children to learn about spatial relationships. We further expect that the relation between the attainment of walking and acquisition of spatial language is mediated by children’s exploration behavior as related to self-locomotion. Spatial language, as measured in the current study, includes locative prepositions (e.g., in, on) and verbs describing movement in a specific direction (e.g., push, climb; Landau and Jackendoff, 1993).

Empirical evidence provides support for a link between attainment of walking and advances in general vocabulary. A previous study with the same sample as in the current study has shown that attainment of independent walking predicted a quicker rate of growth in productive vocabulary between ages 16 and 28 months (Oudgenoeg-Paz et al., 2012). Two other studies have shown that the transition from crawling to walking predicted significant increases in both receptive and productive vocabularies in both the US and China (Walle and Campos, 2014; He et al., 2015). In addition, previous work with the current sample has shown that children who attained independent walking earlier than peers engaged more in exploration through self-locomotion at age 20 months. These children also showed better knowledge of spatial vocabulary at age 36 months. Exploration through self-locomotion at age 20 months, largely mediated this effect (Oudgenoeg-Paz et al., 2015).

Thus, previous work has found support for a link between attainment of walking and general and spatial vocabulary. Moreover, exploration through self-locomotion mediated the relation between walking attainment and spatial language at age 36 months. However, whether these early relations endure over longer time is not yet known. According to the embodied cognition approach the importance of motor development is that it facilitates exploration behavior. Therefore, when examined longitudinally, the initial relations between attainment of walking and linguistic skills are expected to decrease as variability in walking skill decreases as well and most children eventually learn to walk (in the current study all but one child before age 20 months). However, the initial individual differences in exploration behavior are expected to remain important for language development, because exploration provides the embodied conceptual basis for language learning. The mediating role of exploration found in previous work provides support for this hypothesis. As the studies regarding general vocabulary tested the relation between walking attainment and language development close to the onset of walking, it is not yet clear whether these relations also remain when language is measured at later ages. To test this hypothesis, the current study investigated the relation between attainment of walking and receptive general vocabulary and spatial vocabulary at age 43 months, that is at a later time and more distant from the actual attainment of independent walking compared to previous studies. Moreover, direct knowledge of the spatial vocabulary included in the current study (i.e., verbs and prepositions) only develops in the 3rd year of life (Pruden et al., 2004). Therefore knowledge of spatial vocabulary at age 43 months is expected to be more advanced and stable than at age 36 months (the age at which spatial vocabulary was measured in our previous study). To summarize, we hypothesize that the link between walking and general and spatial vocabulary seen at younger ages will be much smaller in magnitude and may even disappear when vocabulary is measured at age 43 months.

Previous studies did not examine the role of exploration through self-locomotion in general vocabulary development. We hypothesize that this kind of exploration is especially important for spatial vocabulary, as the kind of information this exploration provides is relevant for this linguistic domain. Therefore, we do not expect any relation between this kind of exploration and general vocabulary. In our previous work (Oudgenoeg-Paz et al., 2015) we have shown that exploration through self-locomotion mediated the relation between attainment of walking and spatial vocabulary. In the current study we expect to replicate this finding and show that while the initial direct effect of attainment of walking might be smaller (or even disappear completely), exploration through self-locomotion will still mediate the (indirect) relations between attainment of independent walking and spatial language at age 43 months.

If the hypotheses derived from the embodied cognition approach to development are taken too generally, they may imply that motor development is related (through exploration and other possible underlying mechanisms) to all areas of cognitive development. This might be taken by some to simply indicate a general maturation process or broad shifts in general developmental stages. However, the grounding of cognition in real-life experiences suggests that the developmental relationships between motor development, exploration and linguistic skills should be *specific* and intrinsically grounded in the information structures present in the environment and the actions these information structures afford (Thelen and Smith, 1994; Wilson, 2002). For example, exploration of nesting cups is expected to be related to the learning of concepts and words such as *‘in,’ ‘out,’ ‘under,’ ‘on’* in addition to concepts and words related to colors and textures, but not to learning of concepts and words such as different tools used to work in the garden or grammatical knowledge about the use of determiners. Previous empirical work (e.g., Oudgenoeg-Paz et al., 2012; Walle and Campos, 2014) points to linguistic skills that are related to walking attainment, but fails to show which linguistic skills are *not* related to the attainment of walking. Therefore, in the current study, we attempted to distinguish between linguistic skills that are and are not related to motor development. By doing so, the study will contribute important evidence for the distinction between relations based on processes of general maturation and specific relations based on the nature of real-life experiences as suggested by the embodied cognition approach. More specifically, we hypothesize that attainment of independent walking and exploration through self-locomotion will *not* be related to the development of grammatical knowledge. We focus on the use of lexical and grammatical categories including the subject, determiner, auxiliaries and verbal prefix.

Empirical evidence suggests that the use of such lexical and grammatical categories is usually learned from linguistic input provided by the environment (Saffran, 2001). Types of input that seem to be of particular importance for the acquisition of these categories are infant directed speech (Shi et al., 1998), book reading (Naigles and Hoff-Ginsberg, 1998) and conversation eliciting questions in maternal speech (see Hoff, 2006 for a review). As previously discussed, attainment of walking and exploration through self-locomotion are likely to increase naming of objects by parents or prohibiting sentence (e.g., Gogate and Hollich, 2010; Karasik et al., 2011). However, we do not know of any study or theory linking independent walking and exploration through self-locomotion to linguistic input such as infant directed speech and book reading. Therefore, these types of linguistic input are not likely to be elicited by the attainment of walking and exploration through self-locomotion.

To summarize, in the current study three hypotheses were tested: (1) The relation between walking attainment and general and spatial vocabulary measured at age 43 months, will be weaker than the relations reported at earlier ages and may even completely disappear; (2) Exploration through self-locomotion is expected to mediate the (indirect) relation between attainment of walking and spatial language at age 43 months; (3) No relation is expected between attainment of independent walking and exploration through self-locomotion and the use of grammatical and lexical categories at age 43 months. Moreover, no relation is expected between exploration through self-locomotion and general vocabulary.

The current study included 31 Dutch children who took part in a longitudinal study. For the current analyses data were used from home visits at ages 20 and 43 months. Parental reports were obtained about the age of attainment of independent walking. Exploration through self-locomotion was measured using observations of children playing with a standard set of toys at age 20 months. This age was chosen as at this age (almost) all children are expected to be able to walk independently. Therefore, individual differences in exploration at this age do not merely reflect walking proficiency. In addition, in order to compare the magnitude of the relations with previous work, it is important to maintain the same age used in these studies (e.g., Walle and Campos, 2014; Oudgenoeg-Paz et al., 2015) for the predictors and only shift the outcome measures to a later age. Productive spatial language, receptive vocabulary, and command of grammatical and lexical categories were measured at age 43 months using (standard) tests.

## Materials and Methods

### Participants

The sample is a subgroup of participants from a larger longitudinal study. The sample included 31 Dutch children (58% girls). Data of three additional children were excluded, as these children did not participate in the measurement wave at age 43 months. Participants were recruited through day-care centers in the municipality of Utrecht, The Netherlands, and surroundings and through an address list made available by the municipality. Most parents enjoyed medium to high educational and occupational levels. The children had no known developmental disabilities or delays at the time of recruitment. Informed consent was obtained from the parents for all of the children and the study was conducted in accordance with the ethical guidelines of Utrecht University. For the current study data were used from two measurement waves at the age of 20 months (*M* = 20.75, *SD* = 0.61) and at the age of 43 months (*M* = 43.20, *SD* = 0.71).

### Procedure

**FIGURE 1:**
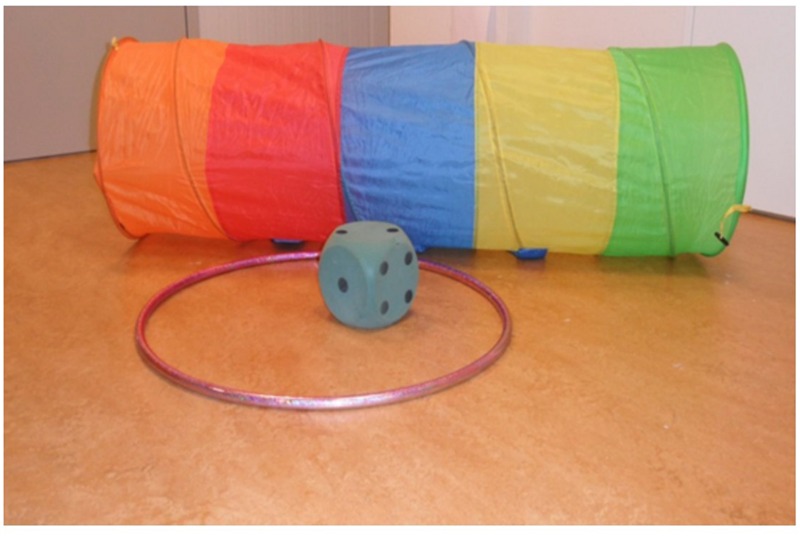
**Objects used in the observations**.

Exploration behavior and linguistic skills were measured during home visits. Exploration through self-locomotion was measured at age 20 months. The children were filmed while allowed to explore a standard set of objects for 8 min. The set of objects included a hoop (70 cm diameter), a large foam dice (15 cm × 15 cm × 15 cm) and a play tunnel made of polyester (150 cm length and 45 cm diameter). See **Figure 1** for a photo of the objects used. The films of children’s interaction with the objects were later edited to remove interruptions (such as stopping to drink). Exploration behavior was then scored based on the first 4 min of uninterrupted play. These 4 min started when the child first made contact with the objects. The duration of 4 min was selected based on pilot coding showing that this duration was both sufficient for most children to interact with all three objects and short enough to prevent children from getting bored and terminating their interaction with the objects. General vocabulary, spatial language and the use of grammatical and lexical categories were measured using playful (standard) tests administered by trained research assistants in a fixed order. The tests of grammatical and lexical categories were administered using a laptop computer. Parental reports were obtained regarding the age of attainment of independent walking. To thank the families for participation, the children were given a small gift at each measurement wave.

### Measures

#### Age of Attaining Independent Walking

At the time of enrolment in the study (between ages 13 and 20 months for the current sample), parents were given the Parental Checklist of Developmental Milestones (Bodnarchuk and Eaton, 2004). Parents were asked to indicate for all milestones in the list at what age their child had attained this milestone. For milestones not yet attained, parents were asked to keep track of their child’s development and note when these milestones were attained. A detailed description of each milestone was provided to help parents decide if their child had attained this milestone. For the current study, parental reports regarding age of independent walking were used. The description of this milestone given to the parents was: “The child is walking unsupported across the room; the child uses walking as the main means of moving around.” Previous studies have shown that parents’ reports using such descriptions are reliable (Bodnarchuk and Eaton, 2004; Berger et al., 2007; Adolph et al., 2011). When the milestone of independent walking was attained prior to enrolment in the study parents were asked to use the records kept by the Child and Infant Health Centre or their own records (such as diaries, blog entries, digital photos, or email communication) to determine the age of onset of independent walking. If parents had no such records, data were considered missing. This was the case for one child in the current sample.

#### Exploration

Children’s exploration behavior was scored by trained observers based on the films of the first 4 min of exploration with the set of objects shown in **Figure 1**. The 4-min recordings were each divided in 24 intervals of 10 s each. Per interval, all the activities of the child and the duration of each activity in seconds were noted. In the current study we were interested in spatial exploration and specifically in exploration through self-locomotion. Therefore, the initial detailed scoring at the level of separate explorative activities (such as banging, carrying, and looking) was aggregated and activities were scored as either stationary (i.e., the child was exploring the objects from a stationary position), or engaged in self-locomotion (i.e., the child was changing location by self-movement, such as crawling or walking while exploring the objects). During the 4-min recordings, most children (over 86%) interacted with the objects for more than 96% of the time. The remaining four children interacted with the objects for a minimum of 20 intervals (i.e., 83% of the time), but most of these children interacted with the objects for longer periods. Thus, the coding represents children’s posture while exploring the objects.

Examples of exploratory actions observed with the objects while in stationary position are: standing and banging on the tunnel, standing in the hoop, sitting in the tunnel, holding the dice and looking at it, holding and manipulating the dice in sitting position, sitting next to the hoop or dice and lifting it. Examples of exploratory actions observed with the objects while engaged in self-locomotion are: crawling through the tunnel with or without the dice, jumping in and out of the hoop, throwing or rolling the dice and running or crawling after it, walking while dragging the hoop or the tunnel, walking around the tunnel while looking at it, lifting the dice and transferring it toward the hoop and putting it into the hoop.

The objects used were all novel for the children and presented various action possibilities to learn about spatial relations. Therefore, all the actions children performed with the objects could be considered as exploration (Weisler and McCall, 1976; Schuetze et al., 1999; see for example also measurement of exploration used by Needham et al., 2002; Lobo and Galloway, 2013). It should be noted that in the current stud we are not referring to locomotor exploration in the sense of navigation and exploring the space, but rather to exploration of the three objects in a way that requires self-locomotion for exploring the spatial affordances of the objects (either separately or as a combination of objects) or for moving from one object to another (see also Cole et al., 2015). For example, when the infant attempts to crawl through the tunnel he or she explores the spatial affordance of ‘moving- through-a-tube’ and learns, among other things, about the size relations between aperture of the tunnel and the body and about the spatial relations of *in* the tunnel and *out* of the tunnel. The exploration of these spatial relations is only possible while engaging in self-locomotion. Similarly, when the infant picks the dice, walks with it and transfers it into the hoop it learns about moving objects in space and changing the spatial location of the dice (i.e., the dice can be lifted, carried and transferred to a different location) and it learns about the size relations enabling the hoop to contain the dice and about the relation of *in* and *out* of the hoop. Again, the learning of these complex affordances requires self-locomotion.

A total score was given to each interval based on the longest enduring activity. If both stationary (scored as 0) and engaged in self-locomotion (scored as 1) were present equally long within an interval, a score of 1 was given to the whole interval. Intervals that could not be scored for technical reasons (e.g., child’s actions were not visible), were given a missing score. About 9% (three children) of the recordings had more than 50% of the intervals missing and were therefore excluded from the analyses. An additional 48% had less than 50% missing intervals and the majority of these recordings (14 out of the 15 children) had less than 25% missing intervals. Two coders independently scored about 22.6% of the films. The mean Cohen’s kappa was 0.81 (*SD* = 0.08) and all kappa values were above 0.70. These values are considered satisfactory (Landis and Koch, 1977).

The total score on exploration through self-locomotion was the proportion of the intervals in which children explored the objects while engaging in self-locomotion at least as much as exploring from a stationary position. This score was preferred to a score based on the total time of self-locomotion, in order to control for the missing intervals.

#### Spatial Language

Productive knowledge of locative prepositions and verbs containing a direction was measured using two playful tasks. Knowledge of propositions was measured using a hand puppet of Ernie from the TV program Sesame Street and a set of small toys. The toys included furniture for a dollhouse and small objects that fit in and around the furniture. The experimenter and the child used the furniture and objects to build a house for Ernie, according to a photo showing where everything should be located in Ernie’s house. The objects were placed so that they represented the entire range of spatial prepositions (e.g., *in* the closet, *between* two chairs). Following this, the child had to explain to Ernie where to find the different objects in the house, by using spatial prepositions to indicate the location of the objects. To prevent the children from pointing to the location, they were asked to talk to Ernie via a toy telephone. The task began with two practice items. During these items the experimenter reminded the children that Ernie cannot see them and that the instruction has to be given in words. When needed, the experimenter modeled the right answer. Children were then encouraged to repeat the right answer. Following the practice items, 10 test items were administered. Each item elicited a different locative preposition. If the child did not answer a question posed by Ernie, the experimenter repeated the question. If the child still did not answer, the experimenter pointed to the location of the object on the photo used to build the house and said: “look there is the [name of object], can you tell Ernie where the [name of object] is?” If the child still did not answer the experimenter provided the right answer. After an answer was given (either correct or wrong, by either the child or the experimenter), Ernie ‘found’ the object and thanked the child. Children were always given positive feedback for providing an answer, regardless if it was right or wrong.

Productive knowledge of spatial verbs was measured using small dolls of Dora and Boots from the cartoon film ‘Dora the Explorer’ and two large (A3) pictures. The pictures each depicted a trail leading to either a beach or a treasure chest. Along the trail different locations and objects were drawn. Dora and Boots were moved by the experimenter along the trail and stopped at each location. Whenever Dora and boots stopped the child was asked what they should do at that location. The answer always included a spatial verb. For example, when reaching a slide, Dora says she wants to go down the slide and asks the child how can she get up the slide. The answer should then include the word *climb*. Also in this test if children did not answer the question, the question was repeated. If the child still did not answer, the experimenter prompted the child by saying for example “how can Dora get up the slide? She has to…” If the child still did not answer, the experimenter provided the correct answer. Children were always given positive feedback for every answer they provided either right or wrong. After an answer was given (either correct or wrong, by either the child or the experimenter) Dora and Boots went on to do the activity and then proceeded on the trail. Each child completed the trails on both pictures. The task included 19 test items representing 19 spatial verbs.

The total score on each task (prepositions and verbs) was the number of words produced correctly. Scores on both tasks correlated strongly (*r* = 0.70, *p* < 0.001). A total score was computed by calculating the mean of the scores on the two tasks after *Z*-transformations were applied. Scores on these tasks correlated moderately (*r* = 0.39, *p* = 0.04 and *r* = 0.38, *p* = 0.07) with scores on a different task measuring receptive and productive knowledge of locative propositions and spatial verbs used at age 36 months (for a description of this task, see Oudgenoeg-Paz et al., 2015).

#### General Receptive Vocabulary

Receptive vocabulary was measured using the Dutch translation of the normed Peabody Picture Vocabulary Test-III (PPVT-III Dunn and Dunn, 2005). In this test the children are shown sets of four pictures each and are asked to point to the picture representing the word said by the experimenter. Each set contains 12 words. Difficulty level of the test varies with age and standard starting and stopping rules are applied. At age 43 months children always start on the third set and testing is stopped when children make nine or more errors within a specific set. Reliability and validity of this test are reported to be good (Dunn and Dunn, 2005).

#### Use of Grammatical and Lexical Categories

The use of grammatical and lexical categories was measured using a sentence repetition task developed by Wilsenach (2006). This task is an elicited imitation task following the rationale that in order for children to repeat a sentence containing a specific structure, this structure should be part of the child’s grammatical skill. The children saw a robot on a laptop screen. The robot said a sentence and the children were then asked to repeat what the robot said. The test included three training items and 12 test items. During the training phase the children were asked to repeat the training sentences. In this phase the experimenter helped the children if needed by modeling the right answer and coaching them in order to avoid use of strategies such as only repeating the last word. The training items could be repeated as often as needed in order for the children to learn the task. In the test phase no coaching was provided. If, during the test phase, a child did not respond or repeated only one word, the stimulus from the robot was repeated once more. If the child repeated only one word also the second time, this was noted as the answer. If the child provided a response (correct or incorrect) a reward was visible. Rewards were various visual effects that appeal to young children, such as balloons flying across the screen. If the child did not provide a response, the next item was presented directly without first presenting the reward.

We used 12 of the 18 sentences included in the original task. In order to adjust the level of difficulty for the current age group we left out the sentences with distransitive verbs used in the original task. The experimenter noted which words were correctly repeated and which were omitted or incorrectly repeated. Scoring included the number of determiners correctly repeated (range 0–23) and the number of subjects, auxiliaries and verbal prefixes correctly repeated (all with a range of 0–12). A Total score was calculated by computing the mean of the scores on all scales after *Z*-transformations were applied. Wilsenach (2006) has shown that this task has good reliability. Items in the test with the current sample also showed good reliability with Crobnach’s alpha of 0.99.

### Statistical Analysis

All three research questions were analyzed using hierarchical regression models, following the steps defined by Baron and Kenny (1986) for testing mediation. First the main effects representing the relation between age of walking attainment and the three dependent variables (i.e., spatial language, general vocabulary and use of grammatical and lexical categories) were tested. Next, the relation between age of walking attainment and the mediator, exploration through self-locomotion, was tested. Third, the mediator was added to the hierarchical regression models. In models where the mediator significantly predicted the dependent variable, the Sobel-Goodman test was applied to test the significance of the mediation. Given the relatively small sample size, bootstrapping was applied in order to obtain more robust parameter estimates.

## Results

### Descriptive Analysis

In **Table 1** the means and standard deviations of all model variables and their indicators are presented. The variables measuring age of walking and spatial language had each missing data from one child. Exploration through self-locomotion and Lexical and grammatical categories had data missing from three children each. Missing data were estimated, where possible, using single regression-based imputation (Schafer and Graham, 2002; Rubin et al., 2007). This method was chosen, rather than the standard listwise deletion, as it has been shown to be an appropriate method for small samples with a low percentage of missing. Moreover, imputing missing data is important with small samples, in order to prevent reduction in power of the analysis (Rubin et al., 2007). The final analyses were conducted with data of 30 children.

**Table 1:** Descriptive statistics of model variables and indicators.

Variable	*N* before imputation	(imputed) *N*	*M*	*SD*
Age of independent walking	30	31	15.24	2.34
Exploration through self-locomotion 20 months	28	30	0.46	0.19
Total score productive spatial language 43 months^a^	30	31	-0.08	0.94
Spatial verbs productive 43 months	-	30	10.63	3.34
Spatial prepositions productive 43 months	-	28	5.83	2.14
Receptive vocabulary 43 months^b^	31	31	56.32	10.05
Total score grammatical and lexical categories 43 months^a^	28	31	0.09	0.94
Determiner omission 43 months	-	28	16.57	5.09
Subject omission 43 months	-	28	9.04	2.70
Auxiliary omission 43 months	-	28	9.89	2.62
Verbal prefix omission 43 months	-	28	11.00	1.19

*Missing values were imputed at the level of total scores, therefore the total scores presented are after imputation but the raw scores still contain all missing values. ^a^This score is a mean of *Z* scores ^b^Raw scores of the PPVT-III were used in the analysis.*

As can be seen from **Table 1** the variable measuring the use of the verbal prefix in the sentence repetition task showed a ceiling effect as the maximum score on this variable was 12. Therefore, this variable was not used in the total score of grammatical and lexical categories. In addition, it should be noted that at age 20 months (the age at which exploration was measured) all children, but one, could already walk for at least 2 months. The child, who could not walk at age 20 months, was able to walk on her knees. **Table 2** presents the correlations between all the variables included in the analyses. **Table 2** shows that the two vocabulary measures (spatial vocabulary and general vocabulary) correlate strongly as can be expected. In addition, the total score of grammatical and lexical categories correlates moderately (though only marginally significant) with the score on general vocabulary, as can be expected. Finally, the indicators of the total score of spatial language correlated strongly with each other (*r* = 0.70, *p* < 0.001). The same was true for the indicators of the total score of grammatical and lexical categories (*r* ranges from 0.74 to 0.87, *p* < 0.001).

**Table 2:** Correlations between all model variables (*N* = 30).

	1	2	3	4
(1) Age of independent walking				
(2) Exploration through self-locomotion	-0.46^∗^			
(3) Total score productive spatial language	-0.06	0.42^∗^		
(4) Receptive vocabulary	0.12	0.20	0.71^∗∗∗^	
(5) Total score grammatical and lexical categories	-0.04	0.14	0.26	0.34^†^

*^†^*p* ≤ 0.10, ^∗^*p* ≤ 0.05, ^∗∗∗^*p* ≤ 0.001.*

### Factors Predicting Spatial Language

To test whether age of walking predicted spatial language at age 43 months and whether exploration through self-locomotion mediates this effect, a hierarchical regression analysis was conducted. The results are presented in **Table 3**. First, the age of walking was entered as a predictor of spatial language. As can be seen from **Table 3**, age of walking was not a significant predictor of spatial language. Next, the relation between age of walking and the mediator exploration through self-locomotion was examined. As can be seen from their correlation in **Table 2**, age of walking was a significant predictor of exploration through self-locomotion at age 20 months. The negative correlation coefficient indicates that an earlier age of walking predicts a higher level of exploration through self-locomotion at age 20 months. Finally, the mediator exploration through self-locomotion was added to the model (model 2 in the top panel of **Table 3**). The results show that this addition leads to a significant improvement of the model and exploration through self-locomotion significantly and positively predicts spatial language at age 43 months. This effect is medium sized. According to Kenny et al. (1998), there can be complete mediation even if the main effect (in this case the relation between age of walking and spatial language) is not significant. This is because the predictor and independent variable might be too far away in time. To test if this mediation is indeed significant the Sobel-Goodman test was performed. Results revealed that, in line with the hypothesis, exploration through self-locomotion indeed completely and significantly mediated the effect of age of walking on spatial language (*Z* = -2.25, *p* = 0.03).

**Table 3:** Results of hierarchical regression analyses for factors predicting spatial vocabulary, receptive general vocabulary and grammatical and lexical categories (*N* = 30 for all analyses).

	Model 1			Model 2			
Predictors	*B (SE)*	β	*R*^2^	*B (SE)*	β	*R*^2^	Δ*R*^2^
**Factors predicting spatial vocabulary**				
Age of independent walking	-0.02 (0.08)	-0.06	0.004	0.07 (0.08)	0.17	0.20^∗^	0.19^∗^
Exploration through self-locomotion				2.47 (0.97)	0.49^∗^		
**Factors predicting receptive general vocabulary**				
Age of independent walking	0.52 (0.81)	0.12	0.12	1.15 (0.89)	0.27	0.10	0.08
Exploration through self-locomotion				17.20 (10.94)	0.32		
**Factors predicting lexical and grammatical categories**				
Age of independent walking	-0.02 (0.06)	-0.04	0.002	0.01 (0.09)	0.03	0.02	0.02
Exploration through self-locomotion				0.74 (1.06)	0.15		

*^∗^*p* ≤ 0.05.*

### Factors Predicting General Vocabulary and Use of Grammatical and Lexical Categories

To test whether age of walking attainment predicted general vocabulary and grammatical and lexical categories and whether exploration through self-locomotion mediated these effects the same steps were followed as in the previous analysis. Two hierarchical regression analyses were conducted and the results are presented in the bottom part of **Table 3**. From **Table 3** it can be seen that age of walking did not significantly predict general vocabulary or the use of grammatical and lexical categories. Moreover, addition of exploration through self-locomotion to the model did not significantly increase the amount of explained variance as exploration did not significantly predict either outcome variable. Thus, in line with the hypotheses, the present study did not find evidence that age of walking attainment predicts general vocabulary or the use of grammatical and lexical categories. Moreover, also in line with the hypotheses, there was no evidence found that exploration through self-locomotion mediates the relation between walking attainment and these outcome variables.

## Discussion

The current study sought to examine whether previously reported relations between age of walking and general and spatial vocabulary are still evident when linguistic skills are measured at age 43 months. In addition, the study aimed to test whether the relations between age of walking and exploration through self-locomotion are specific for certain linguistic skills and not for others. To do so we studied whether age of walking predicts spatial vocabulary, general receptive vocabulary and use of grammatical and lexical categories at age 43 months and whether exploration through self-locomotion, observed at age 20 months, mediates these relations. The results show that the previously reported relations between age of walking and general and spatial vocabulary indeed disappear when linguistic skills are measured at age 43 months. In addition, we have replicated our previous finding (Oudgenoeg-Paz et al., 2015) showing that exploration through self-locomotion mediates the relation between age of walking and spatial vocabulary. However, unlike our previous work, in the current study the direct relation between age of walking and spatial language was no longer significant. Finally, we found no significant relation between age of walking attainment and exploration through self-locomotion and neither general vocabulary nor the use of grammatical and lexical categories. All these findings are in agreement with our hypotheses.

### Long Term Effects of Attainment of Walking

Previous work with the current sample (Oudgenoeg-Paz et al., 2012) and work done by others (Walle and Campos, 2014; He et al., 2015) have shown that, early in life, attainment of walking is related to *general* receptive and productive vocabulary development. However, these studies measured language development relatively close to the age of attainment of walking. The current study extends this literature by showing that when vocabulary is measured later in life (at age 43 months) the initial relation found between walking attainment and vocabulary disappears. Similarly we also show that the relation between age of walking and *spatial* vocabulary previously found with this sample at age 36 months (Oudgenoeg-Paz et al., 2015) is no longer present when spatial language is measured at age 43 months. Other studies have shown that at school age there is only a link between motor skills and linguistic skills such as reading and writing in the case of significant motor delays (e.g., Viholainen et al., 2006). Taken together, these results suggest that over longer periods of time the effects of the age of attainment of motor milestones become smaller as most children eventually learn to walk. We do not think, however, that these findings mean that attainment of walking is not important for linguistic skills. At the short term it is clear that attainment of walking propels language development, as is shown by several studies. The decrease in the size of the effect over time is an example of a cascading effect on development. We return to this issue later in this discussion.

Unlike the relation between walking and linguistic skills, we were able to replicate our previous finding regarding the link between exploration through self-locomotion and spatial vocabulary. Exploration through self-locomotion is still significantly related to spatial vocabulary also when it is measured at age 43 months. Moreover, exploration through self-locomotion is also still related to the attainment of walking and thus mediates the initial effect of walking attainment on spatial vocabulary. Our current findings therefore provide additional empirical support for the role of exploration behavior as a mechanism underlying the relation between walking attainment and spatial language. This finding is similar to another study, where infant object exploration, measured using retrospective parental reports, but not the age of attaining motor milestones related to self-locomotion, predicted spatial memory at school age (Oudgenoeg-Paz et al., 2014). These findings suggest that attainment of walking sets in motion a series of processes that in turn contribute to language development. In this case, attainment of walking allows children to explore their environment in new ways and to extend the embodied knowledge basis that underlies language acquisition. Children that walk independently are able to move around and therefore to change their own location and perspective. They are also able to change the spatial arrangement of the environment. These enhanced exploration possibilities are especially related to walking, rather than to other forms of self-locomotion such as crawling, as the visual information acquired through walking is fundamentally different than the information acquired through crawling (Kretch et al., 2012). Exploration through walking enables children’s learning about spatial concepts and eventually facilitates advances in spatial language, as shown in the current study.

Taken together, the results support the idea of a developmental cascade. Attainment of walking is important initially, as it facilitates ways of interacting with the environment which are important for linguistic development. The mechanisms through which walking propels the development of general vocabulary have not been studied in the current study. Possible underlying mechanisms discussed in the literature are an increase in gestures following the attainment of walking (for a review see Iverson, 2010) and changes in social interaction patterns which bring along changes in linguistic input (Gogate and Hollich, 2010; Clearfield, 2011; Karasik et al., 2011; Walle and Campos, 2014). Whatever the mechanisms are, the current study suggests that in the longer term, the initial relation between walking and linguistic skills diminishes and it is these underlying mechanisms that remain the important predictors of linguistic skills.

### Specific Relations

According to the embodied view of development, the relations between motor skills, exploration and linguistic skills are specific as linguistic skills are grounded in specific sensorimotor interactions with the environment providing specific information. Our findings support this idea. We have shown that attainment of independent walking and exploration through self-locomotion are not related to all areas of linguistic development. Rather, their relation with spatial language (and the relation between walking and general vocabulary found at younger ages) is specific. Information obtained through exploration through self-locomotion, such as information about spatial relations in the larger space, is highly relevant for spatial language, but not for other domains of language.

Traditional approaches to cognitive development view relations between developmental domains as reflecting general maturation or some ‘general developmental factor.’ This general factor would explain why some children develop quicker than others. Some suggest that this domain general mechanism might involve maturational processes, processing speed, cognitive processes such as statistical learning, executive functions or environmental factors (for a review, see Rhemtulla and Tucker-Drob, 2011). Should such domain general mechanism underlie the relations found in the current study, one would expect a relation between the predictors (age of walking and exploration through self-locomotion) and all linguistic skills measured. However, the results seem to favor a situated model of cognition, as presented by the embodied-cognition approach. In this model, language (and any other cognition) is softly assembled in real-time from concrete real-life sensorimotor experiences (Thelen and Smith, 1994; Hockema and Smith, 2009). Therefore, relations between developmental domains are highly specific and intrinsically related to the specific types of information acquired through interaction with the environment.

An additional alternative explanation might be found in cross-sectional relations between motor and exploration skills and linguistic skills at age 43 months. Some studies indeed report such relations (e.g., Hill, 2001; Alcock and Krawczyk, 2010). The argument might be made that early motor and exploration skills predict current motor and exploration skills and these current skills, in turn, are related to current linguistic skills. In the present study no concurrent measures of exploration and motor skills were included. However, a cross-sectional relation between motor and exploration skills does not preclude a longitudinal relation nor contradicts the idea of a developmental cascade. Moreover, the decrease in the strength of relations between walking and linguistic skills implies that the experiences facilitated by the attainment of walking early in life, are the ones that, in turn, facilitate linguistic development.

### Strengths and Limitations

The main limitation of the current study is the small sample size. However, based on previous work (e.g., Oudgenoeg-Paz et al., 2012; Walle and Campos, 2014) we expected to find medium to large effects. The current sample had sufficient power to detect such effects (Kelley and Maxwell, 2003; Tabachnik and Fidell, 2007). Moreover, the use of bootstrapping enabled us to obtain robust estimates despite the small sample size. A second limitation is the measurement of exploration behavior. While this is a good measure of the amount of self-locomotion in general, it does not differentiate between different types of self-locomotion such as crawling and walking. Given recent evidence suggesting that the type of visual information obtained from crawling is essentially different from that obtained through walking (Kretch et al., 2012), it would be interesting to code exploration through self-locomotion also in terms of type of self-locomotion in future studies. The question might also arise whether this measure is not confounded with attainment of walking. It is reasonable to assume that children who attained walking can engage more in exploration through self-locomotion (although crawling children can, of course, also engage in such exploration). However, we measured exploration at the age of 20 months, when all children, but one, were already walking for at least 2 months. Thus, while all children could engage in exploration through self-locomotion, children who attained walking at an earlier age more often chose to explore the objects while engaged in self-locomotion. Furthermore, the correlation between walking attainment and exploration through self-locomotion is medium sized, suggesting that while early walkers do engage more in exploration through self-locomotion at 20 months, other factors also play a role in determining the level of exploration at this age. This measure of exploration also forms a strength of the current study by being relatively context free. That is, while children’s exploration was influenced by the specific context of the objects used, the coding is at the level of position and can therefore be also applied to other contexts using different objects. This will enable future studies to examine if the relations reported in the current study are also found in different contexts. An additional strength of the current study is the fact that the measurements were conducted at the children’s home. While this offered less opportunity for standardization of the measures, it contributes to the ecological validity of this study, as compared to other work done in a lab setting. Finally, the use of multiple methods (parental reports, tests and observations) and the longitudinal design enabled us to study development over multiple domains and test hypotheses pertaining to developmental relations over time.

### Future Directions

Future studies should further explore the specific and intrinsic relations between motor skills, exploration and language development. For example, studies could examine the different aspects of spatial language separately, rather than as a single one-dimensional skill as was done in the current study. An interesting question if whether the same pattern of results is found when verbs and prepositions are studied separately and if the same pattern of results will be found for all spatial words if these are considered individually. Similarly, a more detailed analysis of the general vocabulary data could also be interesting. In the current study, and in most studies in the field, general vocabulary is treated as a single one-dimensional skill. However, if the words in a test such as the PPVT are divided in subgroups representing for example verbs, nouns, prepositions and so forth, hypotheses regarding specific relations between certain linguistic categories and certain motor skills and forms of exploration could be tested. Another interesting direction is the study of the early predictors of grammatical development. Some work suggests that grammatical categories are learned early on through several types of language input such as book reading or asking questions that prompt conversations (Naigles and Hoff-Ginsberg, 1998; Hoff, 2006). The input is, however, not completely independent of the child. Children elicit certain kinds of input through their own actions on the environment (for a discussion of this idea, see Gogate and Hollich, 2010). Therefore future studies should seek for the aspects in children’s interaction with the environment that are likely to elicit input that is relevant for the learning of certain grammatical forms.

## Conclusion

The findings of the current study provide support to several hypotheses derived from an embodied view of development. First, we show that relations between attainment of walking and spatial and general vocabulary that are found at young ages decrease and even disappear with time. We also replicate previous findings showing that exploration through self-locomotion remains an important mediator of the relation between age of walking attainment and spatial language. Thus, results support the idea of cascading effects. While initial differences in motor skills are important for linguistic development early in life, over time individual differences in exploration behavior (which themselves are predicted by differences in the age of walking attainment) seem to be the important predictor of spatial language. Second, the results reveal that the relations between age of independent walking, exploration through self-locomotion and the linguistic skills included in the current study are specific as they were found to be limited to spatial language. This pattern of specific relations supports the embodied-cognition idea of situated language learning in which multiple real-life interactions with the environment provide the semantic basis for learning language.

## Author Contributions

OO-P took the leading role in conception and design and acquisition of the data. She also fulfilled the leading role in analysis and interpretation of the data. She also took the leading role in drafting and revising the article. MV participated in conception and design of the data and had a large role in analysis and interpretation of the data. He also took part in drafting and revising the article. PL participated in conception and design of the data and had a large role in analysis and interpretation of the data. He also took part in drafting and revising the article.

## Conflict of Interest Statement

The authors declare that the research was conducted in the absence of any commercial or financial relationships that could be construed as a potential conflict of interest.
